# Microbiome in cancer metastasis: biological insights and emerging spatial omics methods

**DOI:** 10.3389/fcimb.2025.1559870

**Published:** 2025-06-04

**Authors:** Marianne Meyers, Charlotte B. A. Stoffels, Gilles Frache, Elisabeth Letellier, Maureen Feucherolles

**Affiliations:** ^1^ Department of Life Sciences and Medicine, Faculty of Science, Technology and Medicine, University of Luxembourg, Esch-sur-Alzette, Luxembourg; ^2^ Luxembourg Institute of Science and Technology (LIST), Belvaux, Luxembourg

**Keywords:** cancer metastasis, microbiome, spatial omics, host-microbiome interactions, tumour microenvironment

## Abstract

The role of the microbiome in cancer metastasis has emerged as a critical area of research, with growing evidence suggesting that microbial composition and interactions within the tumour microenvironment may significantly influence metastatic progression. This review explores the role of the microbiome in cancer metastasis, as well as potential key bacteria and their mechanisms through which they could impact tumour dissemination, seeding and growth. Biological models used to study metastasis are discussed to provide context for the further investigation of these interactions. In order to answer unresolved questions regarding the microbiome’s involvement in metastatic dissemination, recent advancements in spatial biology techniques are examined, including spatial genomics, transcriptomics, proteomics and metabolomics, which enable the spatial mapping of microbial interactions within the tumour microenvironment. Additionally, multimodal-omics imaging approaches are highlighted for their potential to integrate multiple molecular layers, offering comprehensive insights into the microbiome’s role in cancer metastasis. The review also addresses the challenges and limitations of these techniques, underscoring the complexity of studying microbiome-tumour interactions and offering directions for future research to better explore and target the microbiological landscape in metastatic cancer.

## 1 Introduction

It is predicted that there will be over 35 million new cancer cases in 2050, a 77% increase from the estimated 20 million cases in 2022 (Bray et al., 2024). Of these, metastasis has been described to be the “ultimate and most lethal manifestation” of cancer (Gerstberger et al., 2023). It is the stage of the disease that places the most significant burden on cancer patient survival, as only a few treatment options are available across all cancer types (Gui and Bivona, 2022). Metastasis can be characterised as an accumulation of genetic and epigenetic changes that allow a cancer to spread from a primary tumour site to distant organs. The spreading of tumour cells, also referred to as circulating or invasive tumour cells, is far from straight forward, having been found to be able to travel through either the circulatory or lymphatic system (Leong and Witte, 2024), or directly invade neighbouring structures (Gerstberger et al., 2023). Additionally, two models of metastatic evolution have been proposed that present challenges for the clinical treatment of cancer metastasis. Firstly, the long-standing belief that metastasis forms in a linear evolution, meaning that only after the primary tumour has progressed to later stages do the cells become invasive and pose a risk of metastasis formation (Gui and Bivona, 2022). However, a phenomenon of parallel evolution has recently been observed in which it is noted that metastatic spread can also occur at earlier stages of the disease than previously thought. This would then mean that the metastatic tumour may develop in parallel and partially separately to the primary tumour (Klein, 2009). Secondly, the phenomenon of metastatic dormancy, which occurs when the disseminated tumour cells that have colonised a distant organ survive in a dormant state. This means that they no longer actively proliferate, and thus often go unnoticed. However, when the conditions become favourable, these dormant cells can regain their proliferative phenotype (Neophytou et al., 2019).

**Figure 1:**
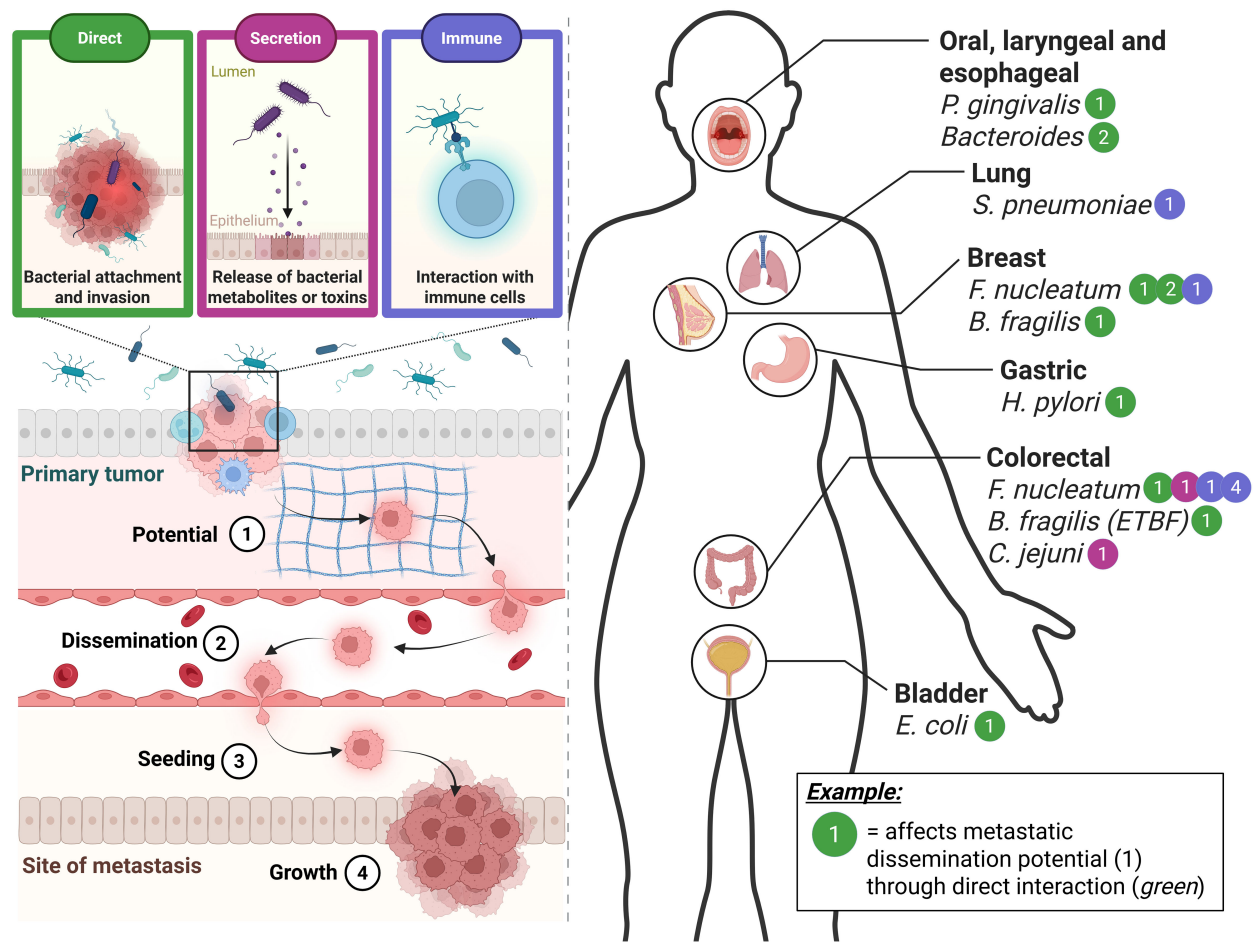
An overview of bacteria suggested to be involved in various stages of metastatic dissemination and cancer types. The varying microbiome-cancer interaction types are represented in the top left and indicated by different colours. The simplified stages of metastatic dissemination are indicated in the bottom left of the figure and denoted by the numbers 1 to 4. A systematic review of the current literature in terms of the different bacterial species suggested to be involved per cancer type is represented on the right of the figure. As an example, it is suggested that Fusobacterium nucleatum, a well-known cancer-associated bacterium, interacts with colorectal cancer in several ways and at several different stages. One of which is that it is known to affect the metastatic dissemination potential (1) via the direct interaction between bacterium and CRC cell (green). Created in BioRender (https://BioRender.com/q18n156).

For the purpose of this review, we have condensed the complex metastatic cascade into four main steps: **1. dissemination potential of the primary tumour, 2. dissemination of invasive tumour cells, 3. seeding of cancer cells at a distant site and 4. growth of the metastasis** (**Figure 1**). Each of these steps is dependent on the tumour cell being able to adopt the different phenotypic cell states necessary to sustain the surrounding tumour microenvironment (TME), including the stromal compartment, and evade the immune system and biophysical properties such as pressure (Quail and Joyce, 2013). Several factors have been identified to play a role in contributing to or shaping the tumour cells’ ability to adopt the different phenotypic cell states and thereby the metastatic spread. The main factors include, but are not limited to, genetics, epigenetics, the immune landscape and the TME (stroma). However, it is the microbiome, in particular, that has recently been gaining traction.

There is growing consensus that cancer is frequently associated with a dysbiosis, defined as an imbalance or deviation from a normal, “healthy” microbiome. While this disruption has traditionally been attributed to alterations in the gut microbiome, recent research has also identified dysbiosis within the local TME across a range of malignancies (Greenhalgh et al., 2016; Coggshall et al., 2019; Liu et al., 2020). It is now suggested that several cancer types—including, but not limited to, breast, lung, ovarian, pancreatic, melanoma, bone, brain, and colorectal cancers—harbour distinct microbiomes. Yet the extent to which these microbiomes are polymorphic, and involved in cancer progression, remains to be explored (Nejman et al., 2020; Battaglia et al., 2024). Nonetheless, Hanahan’s hallmarks of cancer was updated in 2022 to include “polymorphic microbiomes” (Hanahan, 2022). This has led to increased interest in gaining insights into the potential interactions between the microbiome and cancer, with the goal of being able to develop microbiome-based therapies.

Despite being a relatively new field in cancer research, much progress has been made in understanding the intricate interactions between the microbiome and primary cancer (Ternes et al., 2020). While the microbiome includes yeasts, fungi and viruses—each of which has been implicated in tumour progression (Saftien et al., 2023; Mjelle et al., 2025)—this review is focused specifically on the bacterial component of the microbiome. When exploring microbiome-tumour interactions, the interaction types can be approximately classified into three types: **1. direct attachment and/or invasion, 2. indirect secretion of metabolites or toxins, and 3. indirect interaction through the immune system** (**Figure 1**) (Ternes et al., 2020). Direct interactions could include attachment and/or invasion, which could alter tumour cell signalling or downstream phenotypic changes such as cytoskeletal rearrangement (Fu et al., 2022). Alternatively, it has been demonstrated that the secretion of metabolites or toxins from tumour-associated microbes themselves could influence cancer progression (Pleguezuelos-Manzano et al., 2020; Ternes et al., 2022). And lastly, the bacteria can indirectly affect tumour progression through interacting with it and thereby altering the immune system (Xu et al., 2021). However, much less is known about these interactions of the microbiome with metastatic stages of cancer.

In this review, we will explore: (1) the interactions of the microbiome in cancer metastasis identified thus far and how this influences disease progression mechanistically, (2) related biological models for the study of these microbiome metastases crosstalk and (3) subsequent analysis of methodologies based on the principle of spatial omics, which can be used to investigate the interactions between metastases and the microbiome and answer the outstanding biological questions regarding the microbiome metastasis crosstalk. We thereby aim to shed light on how to better explore this evolving, heterogeneous, systemic disease, and the involvement of the microbiome, thus instilling new approaches in the field.

## 2 Biological aspects of the microbiome in cancer metastasis

### 2.1 The influence of the microbiome on cancer metastasis

The breakthrough discovery that bacteria are able to invade into and co-migrate with them during dissemination has evoked a whole new approach to viewing the involvement of the microbiome in cancer metastasis (Bullman et al., 2017). A recent pan-cancer analysis study highlighted the vast variety of tumour resident microbes and their involvement in metastasis. Through integrated *in silico* analyses, they explored the presence of the tumour resident microbes in 4,160 metastatic tumour biopsies, thereby identifying an organ-specific tropism of microbes, and were able to demonstrate that the microbial diversity within the metastasis altered the local immune infiltration, as well as the response to immune checkpoint blockade treatment (Battaglia et al., 2024). Furthermore, a certain spatial organisation of the microbiome within the tumour and TME has been demonstrated, which appears to be highly organised and affect both the immune and epithelial function (Galeano Niño et al., 2022). Interestingly, a study by Fu and colleagues on metastatic breast cancer showed that eradicating the intratumoral microbiome at the primary site *in vivo* actually alleviates metastatic formation (Fu et al., 2022), similarly, Li and colleagues showed that a disrupted gut microbiome, also increased metastasis in a different context of CRC (Li et al., 2019), demonstrating the vital role of the microbiome in the metastatic cascade (Rosean et al., 2019). It has been suggested that the microbiome can affect all the aforementioned stages of metastatic disease (dissemination potential, dissemination, seeding and growth, as summarised in **Figure 1**) by influencing the cancer cell’s ability to overcome the constraints of the extracellular matrix, sheer stress, anoikis and immune surveillance (Fu et al., 2023). In **Figure 1**, we have summarised the current literature concerning the potential involvement of the microbiome in cancer metastasis (**Figure 1**).

One of the most widely studied cancer-related bacteria is *Fusobacterium nucleatum*, with its overabundance being noted in an array of cancers, including oesophageal, pancreatic, breast, and most notably, colorectal cancer (CRC) (Alon-Maimon et al., 2022). It was one of the first bacteria to be identified as travelling together with disseminated cancer cells (Bullman et al., 2017), and is best known for inducing epithelial mesenchymal transition (EMT) in the primary tumour, a vital process in the first step of metastatic spreading (Rubinstein et al., 2013; Kong et al., 2021). It has been shown that *F. nucleatum* invasion into primary breast cancer cells can help the disseminated cell overcome the sheer stress of travelling through the circulatory system (Fu et al., 2022). Not only is *F*. *nucleatum* known to interact directly with cancer cells through attachment and/or invasion, but in the context of CRC, it has also been demonstrated to be able to induce metastatic potential by upregulating the invasive capacity of the primary cells via one of its secreted metabolites, formate, and altering the tumour immune system (Ternes et al., 2022). In breast cancer, *F. nucleatum* was found to suppress the accumulation of tumour-infiltrating T cells at the primary tumour site, thereby contributing to metastatic potential (Parhi et al., 2020). Similarly, in CRC, *F. nucleatum* was found to promote CRC metastasis by affecting M2 polarisation at the primary site (Xu et al., 2021). However, this also occurs at the site of metastasis, the liver, in the case of CRC, where *F. nucleatum* was shown to lead to a lower density of CD8+ T cells (Sakamoto et al., 2021). In general, it has become widely accepted that *F. nucleatum* may play a central role in orchestrating metastatic dissemination, while also being known that it is not the only one and potentially does not act alone.

Another well-established cancer-associated bacterium is enterotoxigenic *Bacteroides fragilis* (ETFB). In breast cancer, it has been found that direct interaction between ETFB and primary tumour tissue potentiates metastatic spread via the activation of Notch and β-catenin axes (Parida et al., 2021). Moreover, in CRC it also affects the potential of primary cancer cells to metastasise via c-Myc expression (Wu et al., 2003). Furthermore, a well-known pathogen that has been associated to CRC, *Campylobacter jejuni*, has recently been found to increase the CRC metastatic potential by activating the JAK2/STAT2/MMP9 axis via its secreted toxin, cytolethal distending toxin (CDT) in the primary tumor site (He et al., 2024). There are also organ-specific cases, such as the well-known gastric cancer-associated bacterium *Helicobacter pylori* which has been linked to EMT in primary gastric cancers on multiple occasions, as observed by Eddin and colleagues (Jamal Eddin et al., 2023). In oral squamous cell carcinoma (OSCC), *Porphyromonas gingivalis* was found to not only induce the migration of cancer cells (via the induction of EMT and MMP secretion) (Lee et al., 2017), but also contribute to the acquisition of stem cell-like properties (Ha et al., 2015), both of which contribute to increasing the metastatic dissemination potential of the primary tumour. Similarly, although remaining at the genera level, a recent study identified *Bacteroidetes* as contributing to the induction of EMT via a TLR4/Myd88/NF-kB axis at the primary site due to lipopolysaccharide (LPS) translocation into the blood in oesophageal squamous cell carcinoma (Wu et al., 2024). In muscle-invasive bladder carcinoma, specific strains of *Escherichia coli* have been linked to EMT signatures (Ha et al., 2015), albeit largely correlatively, suggesting that they play a role in increased metastatic potential. Meanwhile in non-small cell lung cancer, increased metastatic potential was observed after a *Streptococcus pneumoniae* infection (Gowing et al., 2017).

Growing evidence highlights a variety of bacterial roles in metastatic spread, yet fundamental aspects remain largely unknown. For instance, a gut microbiome dysbiosis has been linked to melanoma progression and treatment response (Makaranka et al., 2022; Pal et al., 2022), underscoring the need to understand how remote microbiome can impact metastasis from a distance. Similarly, a disruption of the vascular barrier integrity of the gut has been correlated with bacterial levels in distant metastatic sites (Bertocchi et al., 2021). This raises the question of whether the microbiome plays only a local role at the site of the tumour or whether it can have a systemic effect from distant sites (**local vs. systemic influence**). This approach can also be applied to the microbiome itself. Within the microbiome, certain microorganisms are not uniformly harmful (Makaranka et al., 2022). Some bacteria may drive metastasis, while others might have protective roles, such as the secretome of *Lactobacillus plantarum* YYC-3, which was shown to partially protect against CRC metastatic spread (Yue et al., 2020). Uncovering this balance between harmful and beneficial bacteria is crucial for designing targeted microbial therapies (pathogenic vs. protective microbes). Additionally, microbial species do not act alone. For example, studies show that together, *F. nucleatum* and *P. gingivalis* promote greater cancer cell migration than individually (Lee et al., 2017). Understanding these cooperative dynamics between bacteria could be key to tackling microbial influence on metastasis. Therefore, the crosstalk is not limited to the cancer and bacteria themselves. Bacteria have also been demonstrated to modulate immune responses, as well as potentially impact treatment efficacy. Leveraging this relationship could unlock strategies to strengthen immune-targeted therapies for metastatic disease. These questions demand robust, multifaceted models to capture the dynamic, multi-layered interactions between microbiome and the host. The following section explores the current range of biological models available, assessing their ability to dissect different stages of metastasis and the ways in which they can be used for studying microbiome-host crosstalk. This is followed by a section shedding light onto the new and upcoming techniques that could be used to help answer these outstanding questions of the role of the microbiome in cancer metastasis.

### 2.2 Biological models for the study of metastasis

**Figure 2:**
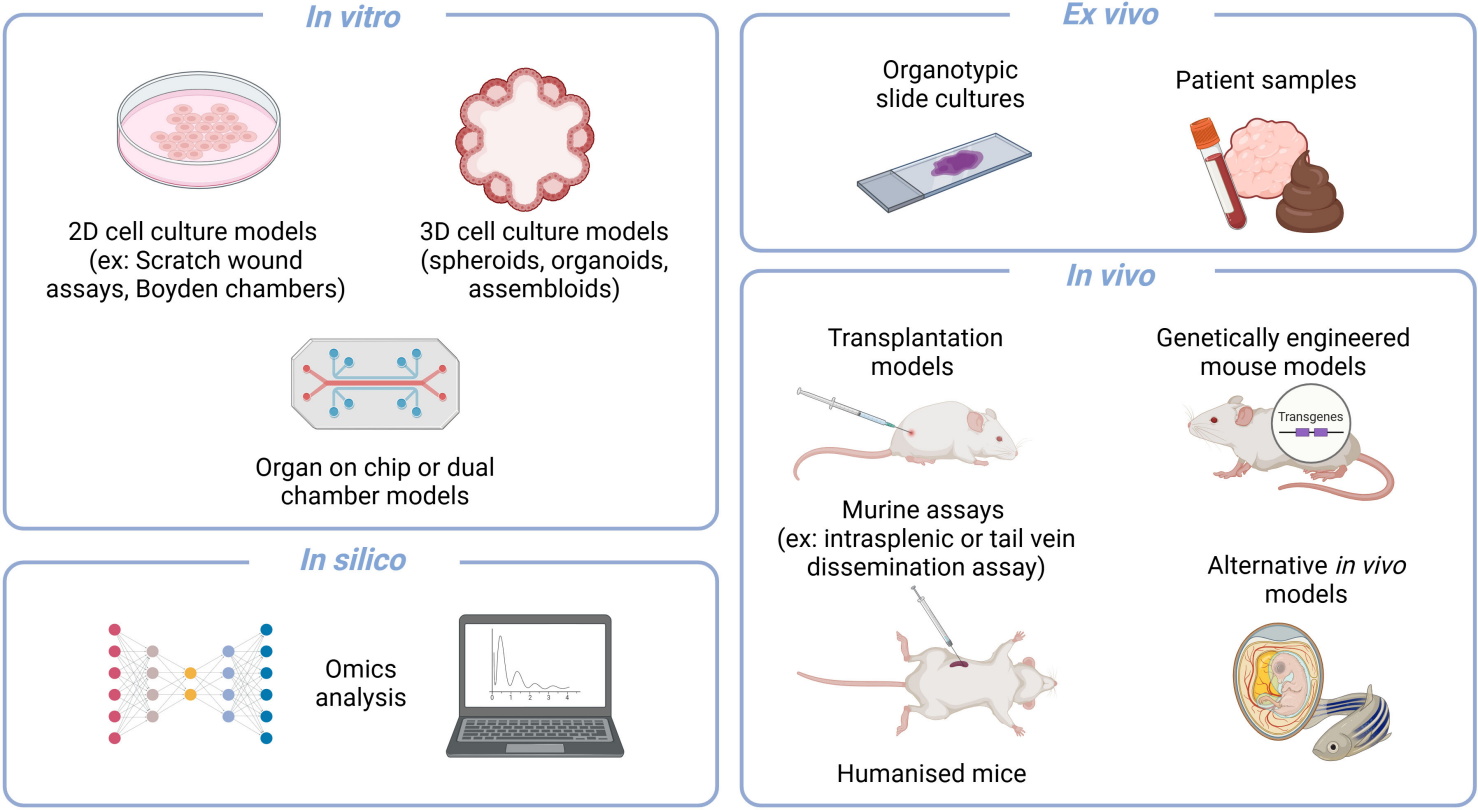
An overview of some biological models that can be used to study aspects of metastasis. Models can be largely classified into the overarching types of *in vitro*, *ex vivo*, *in vivo* and *in silico*. *In vitro* assays include 2D and 3D cultures that can be used in a variety of assays as well as adapted to organ on chip or dual chamber assays. *Ex vivo* setups such as organotypic slide cultures and the analysis of patient samples are valuable approaches that allow for patient relevance. The gold standard remains the variety of *in vivo* murine models that are available for capturing the whole cascade or specific aspects with certain assays. And lastly, the recent vast advances in *in silico* analyses have also allowed it to also become a pilar of scientific exploration. Created in BioRender (https://BioRender.com/q18n156).

The complex process of cancer metastasis is often not only multicellular but also multicompartmental, consisting of several factors and environments at different organ sites. The biological models currently available for the study of this complex, multi-component disease vary from easier yet simplified *in vitro* models, to complex yet difficult to study *in vivo* models (some of them highlighted in **Figure 2**). Each has its own restrictions in terms of cost, time, ethical considerations, complexity, and thus throughput and insights gained (Bouchalova and Bouchal, 2022). The models range from those that provide insights into specific steps and interaction types to those that capture the whole metastatic cascade, yet making it difficult to explore each specific step in mechanistic detail.

*In vitro* models provide the advantages of being relatively low in terms of cost, time and ethical considerations, and thus potentially high throughput. However, the complexity and aspects of the cascade that they can capture are limited. To date, *in vitro* models have been mainly applied for the study of the initial step in metastasis, the dissemination potential of the primary tumour, namely cell migration, invasion, EMT signatures and their respective interactions with the extracellular matrix (ECM). *In vitro* models can vary from 2D and 3D cell culture systems, which can range from simple setups such as the Boyden invasion chambers to assess the invasive capacity of cells (Falasca et al., 2011), to more complex setups such as organ-on-chip systems that allow for the study of more physiologically relevant factors such as movement, gas and nutrients (Zervantonakis et al., 2012; Bersini et al., 2014; Ruiz-Espigares et al., 2021), as well as the combination of several organ sites (Liu et al., 2019; Lucchetti et al., 2024). Furthermore, when the study of the role of bacteria in metastasis is added to these *in vitro* models, they become increasingly complex. Considerations need to be made in terms of culture media, nutrient and oxygen compatibility, as well as pH and waste accumulation levels (Średnicka et al., 2023). In many cases, the bacteria of interest, such as those from the gut microbiome, are anaerobic and therefore an intricate balance needs to be found between aerobic conditions for the eukaryotic cells and anaerobic conditions for the bacteria. In order to address this, several techniques have been applied, such as limiting exposure time with only short co-cultures, only using the spent medium from the bacterial cultures, or adding oxygen scavengers (Qi et al., 2023). However, none of these are optimal as one could miss the window of interaction when limiting the exposure time or would be working under the assumption that the responsible factor is either secreted in the spent medium or could alter the metabolic state of the cells in the co-culture with the oxygen scavengers. Alternatively, one could use dual chamber systems or oxygen gradient cultures, such as some organ-on-chip systems (Lucchetti et al., 2024; Lee et al., 2024). These microphysiological systems enable targeted study of specific compartments, while also allowing for their combination to better reflect the overall biological context. As well as addressing the different oxygen and medium requirements in the dual chamber systems, these culture systems can also address the waste accumulation consideration as there is often a continuous flow in such a system. Another (Qi et al., 2023) elegant example of overcoming these considerations is the microinjection of bacteria into the lumen of 3D organoids (Puschhof et al., 2021). In this setup, the bacteria are at reduced oxygen concentrations in the lumen of the organoid (Williamson et al., 2018), and are closer to their physiologically relevant setting (Lee et al., 2024). Thanks to both the dual chamber culture systems and the organoid microinjections, this gap between bacterial and cancer cell growth requirements has been partially bridged. However, this all works under the overarching assumption that we can culture and work with the bacteria of interest (Średnicka et al., 2023), something which remains a highly limiting factor of bacteria that have been explored in the context of disease.

*An in vivo* approach allows for a broader capture of the metastatic process, including the intravasation up to the colonisation of distant organs. However, it is costly, often requiring long experiments with high ethical considerations. Despite a diverse range of *in vivo* models being available for the study of metastases, the gold standard remains murine models, within which there are several variations. Some capture the whole metastatic cascade, while others are focused on a specific step. Some allow for the capture of all environmental factors including the immune system (syngeneic), while others need to be performed in immunocompromised mice (allogeneic). The disease can be introduced to the model through injections, either orthotopically or via a xenograft, or they can form “naturally” through the use of genetically engineered mouse models (GEMMS). Here, the best large-scale example is the MMTV-PyMT breast cancer metastasis model (Attalla et al., 2020). Alternatively, certain aspects of the metastatic cascade such as the seeding potentially can be studied using specific experimental setups such as intrasplenic or portal vein injections to mimic metastatic dissemination from the pancreas or colon to the liver (O’Brien et al., 2023). Likewise, general tail vein injection assays or iliac artery injections have been used to better mimic intra-venous dissemination and thereby assess seeding capacity (Zhang et al., 2021; Ternes et al., 2022). This model has the advantage of still having all the factors that contribute to the TME that may play a responsible role while focusing on just one step of the whole cascade.

In the context of *in vivo* models, the introduction of the microbiome or single bacteria becomes slightly easier as the physiological context of growth for the bacterium is closer to their natural habitat (Campisciano and Biffi, 2022). Depending on the murine model context (specific pathogen free (SPF) or gnotobiotic/germ-free) and the engraftment potential of the bacterium in the disease context, it may or may not be necessary to pre-treat the mice with antibiotics to allow for stable bacterial engraftment (Campisciano and Biffi, 2022). Alternatively, in specific assays such as the tail vein dissemination assay, cells used for the *in vivo* experiment could simply be pre-exposed to the bacterium to mimic infection prior to the assay (Ternes et al., 2022). The murine models are more complex in terms of the aspects of disease they capture, and including the microbiome aspect. Often, using an SPF background of a complex microbiome can generate a lot of noise in the data, making it difficult to infer specific mechanistic insights. Therefore, researchers have tried to use controlled communities of bacteria, such as the Oligo-Mouse-Microbiota 12 (OMM12), a simplified model of the gut microbiome, with characterized metabolic and ecological interactions, to allow for host-microbiome interaction studies (Eberl et al., 2020). Additionally, the gut microbiome of mice has previously been humanised to make the murine model more physiologically relevant to the human disease context (Lundberg, 2019; Tsenkova et al., 2025).

One must also not forget that with recent significant advancements in omics data generation and analysis, *in silico* predictions have become another vital aspect of studying complex biological processes that were not previously available. A promising avenue is the use of computational modelling for studying either the microbiome’s ecological interactions within the TME, or even the metabolic crosstalk between the microbiome and the host through genome scale metabolic models (Greenhalgh et al., 2019; Clark et al., 2021; Ternes et al., 2022). An example of how *in silico* approaches can contribute to gaining better insights is the pan-cancer analysis of over four thousand omics metastasis biopsies that revealed organ-specific tumour microbiome (Battaglia et al., 2024). Likewise, the use of *ex vivo* models such as organotypic slide cultures or patient sample analyses can help to bridge the gap between fundamental research and patient relevance, however, they are difficult to obtain and only represent one snapshot of a very dynamic disease. Each of these models offers a unique insight into different aspects of the metastatic cascade and possesses various advantages and limitations. Often it is best, as well as necessary, to use a combination of models to fully understand the metastatic process in question and thereby evaluate potential therapies. Not only do the biological models themselves have limitations, it is very diffiucult to analyse it has been very difficult to analyse the microbial component technically. However, with advances in spatial biology, new technical approaches are emerging that allow for the precise localisation and co-detection of microbes within the TME. The next sections will focus on these innovative tools, highlighting how they can help map microbial populations and interactions within metastatic sites, providing deeper insights into the outstanding questions regarding the role of the microbiome in cancer metastasis.

## 3 Technical approaches for exploring the microbiome in cancer metastasis

**Figure 3:**
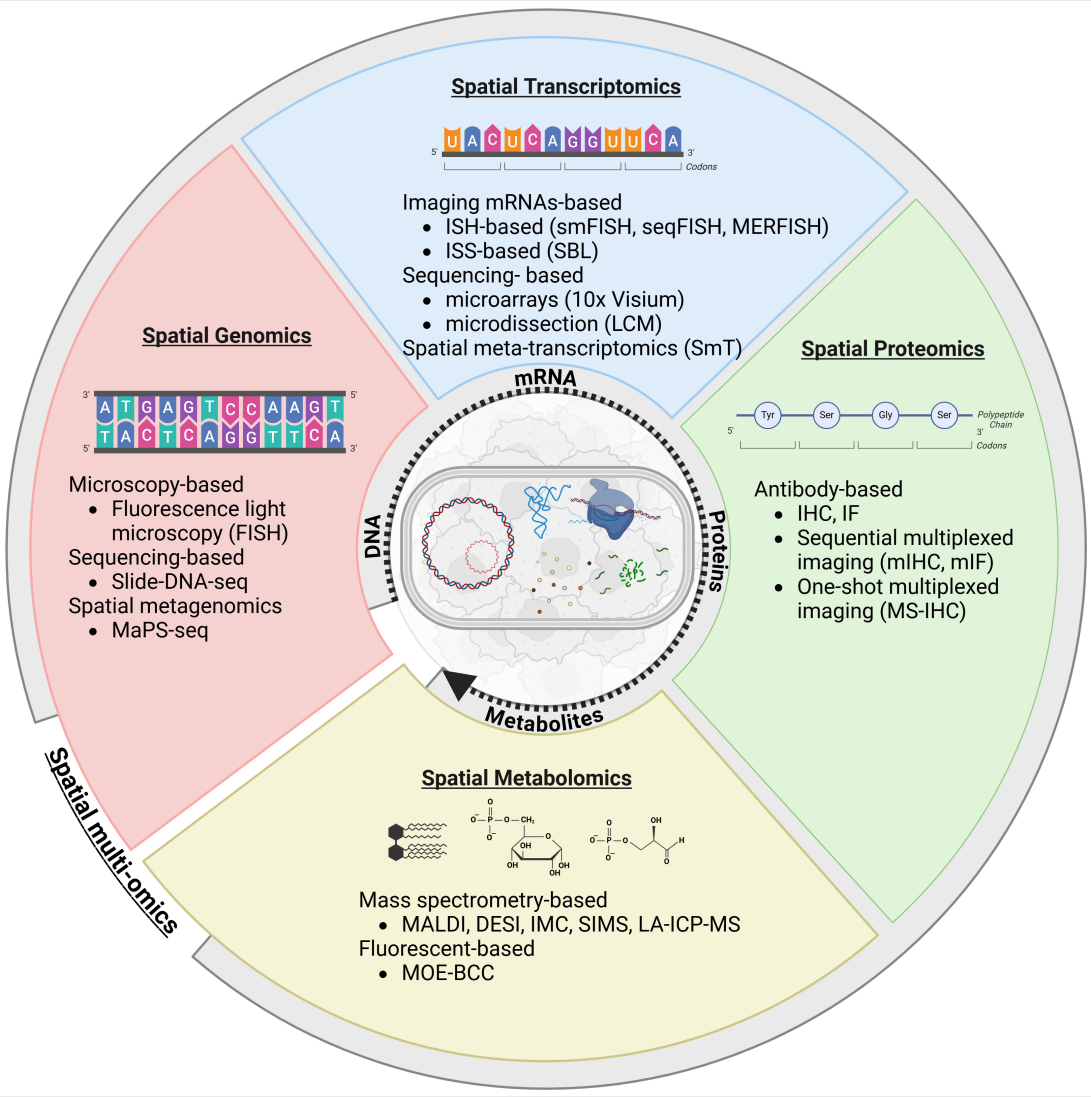
Comprehensive overview of spatial biology applied to the metastasis microbiome to examine spatial interaction between the microbiome and the cancer progression. Created in BioRender (https://BioRender.com/a19q596).

When exploring the microbiome’s potential role in cancer metastasis, bulk omics-powered technology, including genomics, transcriptomics, proteomics and metabolomics, are important as they provide different layers of information that could address current critical questions. For this, conventional techniques are commonly employed, such as whole-genome sequencing, RNA sequencing, mass spectrometry-based proteomics and liquid chromatography-mass spectrometry for metabolomics, applied to bulk samples o specific areas isolated by laser capture microdissection (LCM). However, determining the co-localisation of microorganisms and/or the localisation of biomolecules within specific organelles, cells, or anatomically and histologically defined tissue or organ components presents challenges. Consequently, interpreting omics data poses difficulties, hindering the correlation of overarching metabolic changes with specific tissues, organs or spatially delimited abnormalities, such as tumours or metastases. Recent advancements in spatial biology, driven by next-generation sequencing (NGS), mass spectrometry or fluorescence imaging techniques, have demonstrated efficacy in investigating cell-to-cell interactions and facilitating the visualisation of spatial organisation within cells and tissue structures. These advancements have also made it feasible to map the microbiome and image host-microbiome interactions efficiently and might be directly applicable for metastasis/tumour microbiome research (Bullman et al., 2017; Shi et al., 2021; Galeano Niño et al., 2022; Zhang et al., 2023; Ahkami et al., 2024). The molecular heterogeneity of cancer contributes significantly to drug resistance and treatment failures. Thus, gaining a deeper understanding of cancer heterogeneity holds the key to more precise diagnoses and improved patient outcomes. This knowledge can inform better treatment strategies tailored to individual patients (Hu et al., 2022). The subsequent sections will explore a range of spatial technical (summarised in **Figure 3** and **Table 1**) approaches relevant to deciphering the intricate crosstalk between the tumour microbiome and cancer metastasis environment and elucidate the construction of a microbiological microenvironment tumour or metastasis atlas.

**Table 1:** Summary of spatial omics techniques for investigating the microbiome in metastasis biology.

Omics	Technique	Spatial resolution (pixel size)	Description	Potential applications in microbiome in metastasis	Pros	Cons
**Spatial Genomics**	Fluorescence *in situ* hybridisation (FISH)	~200 nm	Uses fluorescent probes to detect specific DNA sequences in tissue samples.	Localises defined microbial species in tissues, exploring microbial distribution in metastatic niches.	High sensitivity and specificity.	Limited multiplexing, pre-designed probes.
	Slide-DNA-seq	~25 µm	Sequencing-based method that captures DNA in tissue sections for spatial mapping.	Maps tumour DNA heterogeneity in metastasis, associating it with microbial presence.	High genomic resolution.	Limited spatial resolution, potential tissue degradation.
	Metagenomic Plot Sampling by Sequencing (MaPS-seq)	~20-30 μm median diameter	Metagenomics-based approach for sequencing microbial communities in tissue sections.	Identifies and localises microbial communities in metastatic microenvironments.	Broad view of microbial diversity, culture-independent, high taxonomic resolution.	Lower spatial resolution compared to FISH.
**Spatial transcriptomics**	single-molecule FISH (smFISH)	~200 nm	Single-molecule FISH, visualising individual mRNAs with fluorescent probes.	Detects microbial RNA in tumour microenvironments to explore their impact on gene expression in metastasis.	High sensitivity and specificity at single-molecule level.	Limited number of detectable targets per experiment (1–4 genes in a single experiment), fixed samples.
	Sequential FISH (seqFISH)	~100–200 nm	Sequential FISH allowing multiplexing of hundreds of mRNA targets.	Spatial mapping of gene expression changes in metastatic tissues influenced by microbial factors.	High multiplexing capability, single-cell to sub-cellular resolution.	Costly, time-consuming and complex data analysis, errors may accumulate over multiple rounds of hybridisation, targeted approach.
	Multiplexed error-robust FISH (MERFISH)	~100 nm	Multiplexed error-robust FISH, allowing detection of many mRNA species.	Studies how microbial presence and associated gene expression patterns influence tumour cell behaviour, immune response and metastatic survival.	High multiplexing capability, highly robust with minimal error, single-cell to sub-cellular resolution, high-throughput, high sensitivity.	Costly, requires special equipment, high computational demand.
	Arrays-based methods (e.g. 10x Visium)	~2-55 µm	Microarray-based sequencing, capturing RNA with spatial coordinates.	Maps the temporal and spatial dynamics of gene expression in metastasis influenced by microbial presence.	High-throughput, broad tissue coverage, protocols for fresh and fixed samples, compatible with IHC.	Costly, limited spatial resolution, designed primarily for eukaryotic mRNA and requires adaptation for microbial targets, tissue preparation-sensitive.
	Laser capture microdissection-based approaches (e.g. Tomo-seq, GeoMx DSP, Geo-seq)	~5-100 µm	Laser-based microdissection isolates regions for sequencing.	Studies specific tumour and microbial regions in metastatic samples to analyse localised gene expression.	High specificity for targeted areas.	Labour-intensive, low throughput, mRNA degradation during LCM.
	Spatial meta-transcriptomics (SmT)	~55 µm	Meta-transcriptomics focusing on microbial RNA spatial mapping.	Analyses how bacterial and fungal communities evolve during metastatic progression, particularly in response to host immune pressure or cancer treatments.	Captures microbial influence on host gene expression.	Lower resolution than single-cell approaches, risk of capturing environmental contamination signal.
**Spatial proteomics**	Immunohistochemistry (IHC)	~1 µm	Uses antibodies to detect proteins in tissues with chromogenic markers.	Identifies protein markers of immune cells influenced by microbes in metastasis.	Well-established, wide availability, high specificity.	Limited multiplexing, dependency on antibody quality, tissue preparation-sensitive.
	Immunofluorescence (IF)	~200 nm – 1 µm	Uses fluorescent antibodies to visualise proteins in tissues.	Visualises spatial distribution of immune proteins influenced by microbes in metastatic niches.	High sensitivity and specificity, subcellular resolution.	Fluorescence overlap, limited multiplexing, antibody availability, cross-reactivity, limited dynamic range.
	Multiplexed immunohistochemistry (mIHC)	~1 µm	Cyclic antibody staining to visualise multiple proteins in one sample.	Identifies immune markers and microbial influences in metastatic niches.	Higher multiplexing than standard IHC, high sensitivity.	Costly, labour-intensive, longer acquisition times, signal degradation, dependency on antibody quality.
	Multiplexed immunofluorescence (mIF)	~200 nm	Cyclic IF to detect multiple proteins, expanding multiplexing capability.	Identifies how specific bacteria influences metastatic niches, affects the immune response, and contributes to tumour progression.	Higher multiplexing than standard IF, subcellular resolution.	Complexity, costly, fluorescence overlap, potential photobleaching, antibody availability.
	Mass Spectrometry Immunohistochemistry (MS-IHC, e.g. MALDI IHC, MIBI™)	~400 nm - 10 µm	Metal/photocleavable-tagged antibodies used to detect proteins with mass spectrometry.	Characterises the tumour-immune environment, identifies immune-modulatory proteins or neurotransmitters linked or altered due to microbial presence in metastasis.	High multiplexing and sensitivity, cellular to subcellular resolution, no spectral overlap, no background signal, prepared samples are stable indefinitely.	Costly, expensive equipment, complex data processing.
**Spatial metabolomics**	Matrix-assisted laser desorption/ionisation mass spectrometry imaging (MALDI-MSI)	~5-50 µm	Matrix-Assisted Laser Desorption/Ionisation Mass Spectrometry Imaging.	Maps microbial and host metabolites in metastatic tissues, identifies metabolite accumulation related to tumour progression.	No labelling needed, broad range of metabolites, potential for 3D imaging.	Lower spatial resolution, ion suppression effects, less sensitive for low-abundance metabolites, matrix interference and preparation, limited quantitative accuracy.
	Desorption electrospray ionisation mass spectrometry imaging (DESI-MSI, nanoDESI)	~10-200 µm	Desorption Electrospray Ionisation, offering ambient ionisation for MSI.	Rapidly identifies microbial metabolites in metastatic tissues, exploring their roles in tumour invasion and immune evasion.	Minimal sample preparation, ambient conditions, rapid spatial analysis on intact samples, sequential analysis on the same tissue, broad range of metabolites, high throughput.	Limited spatial resolution, less sensitive for low-abundance metabolites, instrument cost.
	Imaging mass cytometry (IMC)	~1 µm	Imaging Mass Cytometry using metal-labelled antibodies for spatial proteomics.	Examines immune cell populations within the tumour microenvironment, providing insights into how microbiome interactions modulate immune responses.	High multiplexing, sensitivity and spatial resolution, minimal signal overlap.	Costly, instrument cost, long acquisition times for detailed mapping, optimised for protein analysis.
	Secondary ion mass spectrometry (SIMS, nanoSIMS, TOF-SIMS, Orbi-SIMS)	~50–500 nm	Secondary Ion Mass Spectrometry for nanoscale analysis of elemental composition.	Visualises microbial metabolic activity and its interactions with tumour cells at a single-cell and sub-cellular level in metastatic niches (or tracing isotopic labels *in situ*).	Extremely high spatial resolution, single-cell level analysis, elemental and isotopic sensitivity.	Instrument cost, skilled operators, complex sample preparation, potential molecular fragmentation, destructive analysis.
	Laser ablation–inductively coupled plasma–mass spectrometry imaging (LA-ICP-MSI)	~1-100 µm	Laser Ablation Inductively Coupled Plasma Mass Spectrometry for elemental imaging.	Visualises metal distribution in microbial regions within metastatic tissue, exploring metal-associated metabolic pathways in the tumour microenvironment.	High sensitivity for trace element, useful for drug-tissue interaction studies, elemental and isotopic analysis, quantitative analysis, preserving tissue integrity.	Limited to elemental analysis, low molecular information, challenging biological interpretations.
	Metabolic oligosaccharide engineering and bioorthogonal click chemistry (MOE-BCC)	~1 µm	Fluorescent labelling of metabolites for imaging biochemical processes.	Studies microbial metabolite spread from primary tumour to metastasis, to understand microbial influence on metastatic development (tumour and metastasis spread).	High specificity, real-time visualisation, dynamic studies.	Requires specific metabolic labelling; limited to detectable fluorophores, dependence on cell metabolism.

This table provides a comparative analysis of spatial omics techniques, highlighting their potential to unravel the complex interplay between the microbiome and metastatic processes, with a focus on spatial resolution, and the strengths and challenges associated with each technique.

### 3.1 Spatial genomics: how do microbial communities spatially organise within metastatic sites, and do specific bacteria exhibit preferential colonisation patterns within metastases?

Understanding how microbial communities are organised within metastatic sites and whether specific bacteria prefer metastatic over primary tumours remains an important open question in cancer research. Additionally, investigating how the spatial distribution of the microbiome evolves during metastatic progression and identifying potential microbial signatures unique to different stages are crucial steps toward deciphering the role of the microbiome in metastasis. Addressing these questions requires technologies that preserve the spatial context of both microbial and host cells within tissues, an area in which spatial genomics is particularly powerful. Spatial genomics combines genomic analysis with spatial information to examine genetic and microbial elements within their native tissue environments. Unlike traditional genomic techniques that lose spatial information, spatial genomics preserves these critical relationships, enabling detailed mapping of microbial communities relative to tumour cells.

#### 3.1.1 Fluorescence light microscopy

##### 3.1.1.1 Fluorescence *in situ* hybridisation

Fluorescence *in situ* hybridisation (FISH) has emerged as a powerful tool to address the questions of microbial spatial organisation within metastatic sites and the potential interactions between microbial communities and host genetic elements. FISH is a powerful molecular cytogenetic technique that visualises and maps the spatial organisation of nucleic acids (DNA or RNA) within cells and tissues (Bayani and Squire, 2004). It involves hybridising fluorescently labelled probes to specific sequences in the target sample, allowing the direct visualisation of these sequences through fluorescence microscopy. FISH is particularly valuable in spatial genomics, where it is used to map the distribution of specific DNA sequences or genomic features within tissue samples. Its ability to use multiple fluorescent colours enables the simultaneous monitoring of multiple genes, making it especially useful in tumour biology, for example, for analysing the localisation and quantification of a specific consortium of bacteria (e.g. Oligo-Mouse-Microbiota, OMM^12^) or mobile genetic elements (e.g. antimicrobial resistance plasmids) in its host (Brugiroux et al., 2022; Grodner et al., 2024). However, FISH has limitations, particularly its dependence on predesigned probes, which restricts its ability to provide comprehensive spatial descriptions of unknown microbiomes, comprising up to thousands of microbial species. Despite this, advancements in FISH technology, such as multiplex FISH, Combinatorial Labelling and Spectral Imaging FISH (CLASI-FISH) (Valm et al., 2012), High Phylogenetic Resolution microbiome mapping by FISH (HiPR-FISH) (Shi et al., 2020), sequential error-robust FISH (SEER-FISH) (Cao et al., 2023), and more recently, mobile genetic elements FISH (MGE-FISH) (Grodner et al., 2024) have significantly expanded the potential of this technique. These innovations have improved the sensitivity, specificity and resolution of FISH, enabling more detailed and accurate genomic and microbial analyses. For instance, Cao and colleagues developed the SEER-FISH workflow, a multiplex approach to mapping microbial communities at the micron scale, based on error-robust encoding schemes. They applied their methodology directly to spatially mapping root-colonised microbial communities of *Arabidopsis* and their relative spatial patterns. More details regarding the operating mode are provided in the original article (Cao et al., 2023). Such an approach might be relevant when investigating short-range interactions (e.g. *quorum sensing*), as well as the attachment and invasion of cancer cells. By leveraging these techniques, the evolution of microbial communities over time during metastatic progression, the identification of specific bacterial species that preferentially colonise metastatic rather than primary tumours, and the exploration of the potential impact of microbial-host interactions on tumourigenesis and metastasis, is possible.

##### 3.1.1.2 Expansion microscopy

Metastasis or the TME may contain a high population of heterogenous cells, including bacteria and immune cells, making it challenging to distinguish between cell species and the specific physiological related states identified. As previously mentioned, FISH is routinely used to visualise targeted DNA sequences of microbes. Nevertheless, such a method is not suitable for investigating physiological changes in bacterial cells that are modulated by their local environment and are thought to be crucial for the microbes’ spatial organisation. To overcome this challenge, the cells were successfully physically expanded prior to imaging. Lim and colleagues introduced the expansion microscopy method (μExM) (Lim et al., 2019), in which embedded fluorescent-engineered bacterial cells, in a swellable polymer matrix, were subjected to a physical expansion process before fluorescent microscopy, leading to an enlargement of the cells and their internal structures. In the end, the authors highlighted the possibility of differentiating species within a defined *in vitro* community of human gut commensals, conducting *in vivo* imaging (e.g. bioluminescence imaging, magnetic resonance imaging, positron emission tomography, ultrasound) of a model gut microbiome, and accurately detecting cell-envelope damage caused by antibiotics, as well as previously unnoticed cell-to-cell phenotypic variability among pathogenic bacteria during macrophage infection. The application of *in vivo* imaging approaches to study host-microbiome interactions is detailed elsewhere (Campisciano and Biffi, 2022). Such an approach could provide valuable insights into the morphology, physiological state and organisation of cells, as well as their interactions with their micro-environment, when in contact with a test molecule.

#### 3.1.2 Slide-DNA-sequencing

Metastasis, as well as tumour heterogeneity, are the results of the accumulation of a variety of DNA mutations or alterations, and extensive chromosomal rearrangement. Therefore, there is a need to develop a workflow enabling the visualisation of both genomic sequences and phenotypic displays. Technologies like Slide-DNA-seq (Zhao et al., 2022) enable a spatial mapping of DNA sequences, directly from intact tissue, by transferring tissue sections to a slide prepared with beads containing a unique DNA barcode that corresponds to a specific coordinate. After sample preparation, the DNA sequencing library is paired and end sequenced. By using the barcode on specific coordinates, the spatial organisation of DNA in tissue can be reconstructed without needing a microscope. In their work, Zhao and colleagues successfully demonstrated that the method accurately preserves sample architecture, and detects clonal heterogeneity and its related localisation (Zhao et al., 2022). This technique could be either used as it is, to provide a genetic map of the microenvironment in which the metastasis microbiome evolves, or the indexed array could be customised to investigate microbial DNA sequences. In any case, and as mentioned by the authors, the slide-DNA-seq may be used in the future for a large-scale study to create atlases of tumour or metastasis evolution.

#### 3.1.3 Spatial metagenomics

Two main drawbacks of using imaging approaches based on the hybridisation of DNA probes (e.g. FISH) to image microbial community, are the (1) spectral diversity limitation, leading to poor taxonomic resolution, and (2) the fact that bacteria are packed in communities, limiting the analysis of individual cells (Welch et al., 2017). Over the last decade, metagenomics has emerged as a powerful tool for investigating the microbial diversity and functions. Metagenomic Plot Sampling by Sequencing (MaPS-seq) (Sheth et al., 2019) is a cutting-edge technique designed to overcome the limitations of traditional methods in characterising complex microbiomes. It suggests a culture-independent solution to map microbial communities with micron-scale resolution, involving the embedding of intact microbiome samples in a gel matrix, cryo-fracturing them into smaller particles and then encapsulating these particles into droplets. Within each droplet, microbial DNA is amplified using barcoded 16S rRNA primers, and the products undergo deep sequencing. This process allows for the detailed spatial and taxonomic profiling of the microbiome, revealing intricate microbial interactions and spatial arrangements that are often missed by other techniques. MaPS-seq thus represents a significant advancement in metagenomics, providing a more comprehensive and nuanced view of microbial communities in complex ecosystems. In this context, MaPS-seq should be applied directly onto metastatic tissues to better understand the spatial organisation of microbiome communities.

All things considered, spatial genomics techniques could be used to study the microbiomes of metastasis because they provide a thorough understanding of the spatial arrangement of genetic features in metastatic tissues, making it possible to characterise both the microbial and host components at the same time. This method could clarify the relationship between a particular genomic feature in the tumour cells and the surrounding microenvironment and the spatial distribution of microbial populations within metastatic lesions. This could provide insights into the role of the microbiome in the progression of metastasis and identify potential targets for therapy.

### 3.2 Spatial transcriptomics: how does a microbial presence within metastatic niches influence cancer cell gene expression and support metastatic survival?

To understand how microbial presence and gene expression within metastatic niches influence cancer cell gene expression and support metastatic survival, it is crucial to examine the complex role that microbes play in shaping cancer progression. The advent of single-cell transcriptomics (scRNA-seq) has revolutionised biomedical research by facilitating the investigation of cellular heterogeneity and gene expression profiles at unprecedented resolutions. However, scRNA-seq is not without limitations, including the necessity for cell isolation procedures that may induce stress, cell death or aggregation, and crucially, the loss of spatial context inherent in dissociating cells from their tissue microenvironment (Li et al., 2022; Williams et al., 2022). In this context, the emergence of spatial transcriptomics (ST) has garnered significant attention, being heralded method of the year in 2020 by Nature Methods (Marx, 2021). ST represents a transformative approach that enables the simultaneous capture of transcriptomic information, i.e. RNA transcripts, and positional context within tissue samples (Marx, 2021). This spatially resolved single-cell analysis holds promise across various disciplines, including cancer research, where it offers invaluable insights into tumour heterogeneity, spatially dependent mechanisms, the tumour immune microenvironment, and pathological classification. By quantifying gene transcripts across distinct spatial locations within tissues, ST techniques provide a nuanced understanding of cellular interactions and spatial organisation, albeit with considerations such as tissue size, number of genes counted and spatial resolution, often necessitating trade-offs. For a long time, the literature on ST was unclear, with numerous techniques and confusing acronyms. However, these techniques have been subdivided into two main groups by other reviewers: imaging mRNAs and sequencing-based spatial transcriptomics (Lewis et al., 2021; Moses and Pachter, 2022; Williams et al., 2022). This categorisation helps streamline the understanding and application of spatial transcriptomics in metastasis microbiome research. A detailed list of existing ST techniques and a comprehensive elucidation of these methodologies can be found in previous literature (Moses and Pachter, 2022; Williams et al., 2022; Cheng et al., 2023).

#### 3.2.1 Imaging mRNAs-based spatial transcriptomics

The imaging of mRNAs *in situ* via microscopy constitutes the foundational principle of imaging-based spatial transcriptomics technologies. This method primarily relies on fluorescence *in situ* hybridisation (FISH), a technique utilised for labelling and visualising mRNAs using fluorescent oligonucleotide probes. This approach encompasses two distinct methodologies: *in situ* hybridisation (ISH)-based and *in situ* sequencing (ISS)-based. ISH-based techniques, such as single-molecule FISH (smFISH) (Femino et al., 1998), entail multiple rounds of hybridisation, with each labelled transcript either appearing as a distinct spot under microscopy, with multiple variants such as sequential FISH (seqFISH) (Lubeck et al., 2014), seqFISH+ (Eng et al., 2019) and multiplexed error-robust FISH (MERFISH) (Chen et al., 2015) or providing enhanced multiplexing capabilities. Commercial platforms, such as Merscope (Vizgen) or CosMX (NanoString) (He et al., 2022), further expand the utility of these techniques. For instance, the SeqFISH method has been adapted for bacterial studies (ParseqFISH), enabling the identification and spatial resolution of heterogeneous metabolic and virulence-related transcriptional states within *Pseudomonas aeruginosa* populations during planktonic growth (Dar et al., 2021). In contrast, ISS-based approaches involve the direct sequencing of amplified mRNAs within tissue sections using sequencing by ligation (SBL).

#### 3.2.2 Sequencing-based spatial transcriptomics

In sequencing-based ST approaches, mRNA molecules are captured from tissue samples, converted into cDNA, before gene-specific sequences are counted via next-generation sequencing (NGS). This category encompasses two main methods: microdissection-based and array-based techniques. Microdissection-based methods involve *laser capture microdissection* (LCM) to isolate regions of interest (ROIs) within tissue sections, enabling transcriptomic profiling via microarrays. Noteworthy developments include *Tomo-seq* (Kruse et al., 2016) and *Geo-seq* (Chen et al., 2017), alongside commercial platforms like Nanostring’s *GeoMx* DSP (Merritt et al., 2020), which offer spatially resolved RNA and protein profiling from distinct tissue compartments. However, limitations include spatial resolution constraints and mRNA degradation during LCM. On the other hand, array-based methods, exemplified by the *Visium* platform from 10x Genomics, provide larger tissue sections and positional barcoding for mRNA capture (Ståhl et al., 2016). Galeano and colleagues used an adapted 10x *Visium* to determine the identity and the *in-situ* location of intra-tumoural microbial communities within patient tissues (CRC and OSCC). With the *GeoMx* DSP, they quantified the expression profile of 77 proteins associated with anti-tumour immunity and cancer progression (Galeano Niño et al., 2022). Recent advancements, such as *Visium* HD Spatial Gene Expression, offer increased spatial resolution, i.e. 11 million features in a continuous grid-pattern of 2 μm squares. Other notable developments include *Slide-seq* (Rodriques et al., 2019), *Slide-seqV2* (Stickels et al., 2021), *High-definition* sp*atial transcriptomics* (HDST) (Vickovic et al., 2019) and *Stereo-seq* (Chen et al., 2022), the latter promising 500 nm spatial resolution, which might be of interest for the purpose of microbiome research. Despite these advancements, array-based methods may not accurately capture cellular morphology contours. Notably, in the context of microbiome research and cancer metastasis, there is a pressing need to elucidate host-microbiome interactions while preserving accurate spatial context (Chatterjee et al., 2024). Emerging techniques like Spatial host–microbiome sequencing (*SHM-seq*) offer all-sequencing-based approaches, enabling simultaneous capture of tissue histology, host transcripts and bacterial 16S sequences (Lötstedt et al., 2023). Studies by Galeano and colleagues, and Lötstedt and colleagues exemplify the utility of these methods in mapping intra-tumoural microbial communities and correlating spatial gene expression programmes with a bacterial presence, underscoring their significance in investigating the metastasis ecosystem and related microbial communities.

#### 3.2.3 Spatial meta-transcriptomics

While ST methodologies are mainly used for investigating the bacterial spatial gene expression over a complex bio-sample, these techniques are unable to obtain bacterial and host transcriptional information simultaneously. Spatial meta-transcriptomics (SmT) is an innovative molecular biology approach that provides a holistic understanding of gene expression patterns within spatial contexts, encompassing both the host and microbial communities, including bacteria, fungi and viruses. Based on modified commercial solutions such as NanoString Digital Spatial Profiler and 10x Genomics *Visium* protocols, SmT has been instrumental in uncovering spatial associations within diverse microbiome ecosystems, from tumours to plant microbiome (Wong-Rolle et al., 2022; Saarenpää et al., 2023). For instance, it has revealed correlations between intra-tumour bacterial burden and lung cancer progression, shedding light on disease mechanisms. Indeed, Wong-Rolle and colleagues highlighted the link between oncogenic β-catenin expression and the bacterial burden, thanks to a method characterising abundance of bacteria (*16S rRNA*), fungi (*28S rRNA*), cytomegalovirus (*UL83*) transcripts and around 1,800 human genes products involved in both cancer and immune pathways, boosting insights into tumour biology (Wong-Rolle et al., 2022). Along the same lines, a multimodal *16S*/*18S*/*ITS*/*poly-d(T)* array capable of characterising host transcriptomes and microbiomes at a resolution of 55 µm was designed by Saarenpää and colleagues, to investigate inter- and intra-kingdom spatial dynamics. They linked local changes in host gene expression to the size and composition of local microbial populations in the model system *Arabidopsis thaliana* leaves (Saarenpää et al., 2023).

Overall, ST could be employed to investigate metastasis microbiomes by providing a comprehensive understanding of the spatial organisation of gene expression profiles within metastatic tissues, thereby enabling the simultaneous characterisation of host and microbial components. This approach could elucidate how the spatial distribution of microbial populations within metastatic lesions correlates with known metastatic genes such as *SOX9* and *SNAI1* (Cai et al., 2024), in both the tumour cells and the surrounding TME, offering insights into the role of the microbiome in metastasis progression and potential therapeutic targets.

### 3.3 Spatial proteomics: how do microbial interactions shape the spatial organisation and immune modulation of metastatic niches?

To understand how the microbiome modulates the immune response in metastatic niches, it is essential to explore the intricate interactions between microbial communities, host cells and the immune system. Recent advancements in spatial proteomics have provided valuable insights into how proteins are organised spatially within tissues, shedding light on the mechanisms by which the microbiome can influence immune cell function and response in these metastatic environments. Spatial proteomics, which maps the localisation of proteins and their interactions within tissues, is key to understanding how immune cells are recruited, activated or suppressed in the presence of microbial signals. Techniques such as immunohistochemistry (IHC), immunofluorescence (IF) and proximity-based assays (e.g. proximity ligation assays) are commonly used to visualise protein localisation and interactions, allowing for a detailed analysis of immune responses in the microbiome context. By examining the spatial distribution of immune-related proteins and their interactions with microbial-derived factors in metastatic niches, spatial proteomics enables the identification of immune modulators influenced by microbial presence. This approach not only deepens our understanding of immune microenvironment and tumour progression, but also highlights potential therapeutic targets to modulate the immune response in metastatic disease.

#### 3.3.1 Antibody-based sequential multiplexed imaging

To understand how the microbiome modulates the immune response in metastatic niches, advanced techniques for detecting and localising immune-related proteins within tissues are essential. IHC and IF are widely used techniques for detecting and localising proteins in cells and tissues by exploiting the specific binding between antibodies and antigens using light and fluorescence microscopy, respectively. Traditionally, antibodies are visualised through enzyme-mediated indirect labelling or direct conjugation with chromogens or fluorophores. However, these methods are limited in the number of markers that can be imaged simultaneously. Emerging technologies, such as multiplexed immunohistochemistry (mIHC) and multiplexed immunofluorescence (mIF), address this limitation by allowing the simultaneous visualisation of numerous proteins within a single tissue section (Taube et al., 2020; Park et al., 2022). mIHC and mIF utilise a cyclic staining approach to analyse the same sample in multiple rounds (e.g. *COMET*™ from Lunaphore). After each staining cycle, image assembly algorithms were employed to integrate the data. Examples of sequential mIF techniques include Cyclic Immunofluorescence (CyCIF) (Lin et al., 2018), which uses repeated cycles of staining and imaging, Iterative Bleaching Extends Multiplexity (IBEX) (Radtke et al., 2020), which involves iterative bleaching to extend the range of detectable antigens, Iterative Indirect Immunofluorescence Imaging (4i) (Gut et al., 2018), Co-Detection by Indexing (CODEX, newly PhenoCycler by Akoya Biosciences) (Goltsev et al., 2018) and Immunostaining with Signal Amplification by Exchange Reaction (Immuno-SABER) (Saka et al., 2019). Unlike traditional methods, Immuno-SABER and CODEX use antibodies conjugated to unique DNA oligonucleotides rather than fluorescent dyes. It is worth noting that recent studies have also shown the possibility of combining multiple techniques, such as mIF and MS imaging (Goossens et al., 2022). Overall, the development of such methods has allowed the simultaneous assessment of multiple biomarkers to study the tumour immune microenvironment. Despite these advances, challenges such as lengthy acquisition times and potential signal degradation remain, which may limit the scalability of these techniques for high-throughput studies. Nevertheless, the ability to assess multiple immune and microbial biomarkers within the tumour-immune microenvironment is crucial for understanding how microbial modulation supports immune evasion and metastatic progression.

#### 3.3.2 Antibody-based one-shot multiplexed imaging

One-shot multiplexed imaging approaches, such as Digital Spatial Profiling (DSP) (Ahmed et al., 2022), Stimulated Raman Scattering (SRS) microscopy (Wei et al., 2017) and Mass Spectrometry Imaging (MSI), produce highly detailed and informative images. However, their widespread use is often limited by the high cost of the consumables and specialised equipment required. Mass Spectrometry Immunohistochemistry (MS-IHC) is an increasingly important method for characterising the spatial organisation of proteins in biological samples using metal-tagged antibodies, allowing for the simultaneous detection of up to 40 different protein markers. Current MS-IHC techniques include Multiplexed Ion Beam Imaging (*MIBI*™) (Keren et al., 2019), developed by IonPath, which utilises a charged primary ion beam, and Imaging Mass Cytometry (IMC), which employs a pulsed laser beam (Giesen et al., 2014; Feng et al., 2022; Kuett et al., 2022), reaching a spatial resolution of 400 nm. In the work of Keren and colleagues, MIBI-TOF technology was used to resolve regional heterogeneity in the tumour-immune microenvironment by analysing the localisation of 36 proteins, including tumour and immune cell phenotypes, and immuno-regulatory proteins, such as vimentin and EpCAM molecules which are highly expressed in metastatic cancer, in single triple negative breast tumours (Spizzo et al., 2011; Keren et al., 2019; Berr et al., 2023).

Similarly, while conventional MSI techniques, such as Matrix-Assisted Laser Desorption/Ionisation (MALDI) MSI, are well known for not requiring prior tagging to detect a broad range of biomolecules, including peptides and proteins, in a single scan without altering the native sample (Feucherolles and Frache, 2022), it is possible to target specific hardly ionisable molecules by using photocleavable mass tags, enabling the simultaneous imaging of several intact proteins (MALDI-IHC) (Yagnik et al., 2021). Commercial solutions like *Miralys*™ from AmberGen propose several panels, such as immune-oncology, tissue morphology, lung and breast cancer, neurological and cancer core, for targeted spatial proteomic studies. Although commercial panels for immune-oncology and cancer studies are available, microbial proteomic panels, particularly for analysing the bacterial microbiome in metastatic niches, have yet to be fully developed using a customised conjugation kit. Such panels could provide essential information on how microbial communities interact with immune cells to modulate immune responses, influencing metastasis progression. Incorporating microbial proteomics into spatial imaging techniques could significantly enhance our understanding of how the microbiome shapes the immune response within metastatic microenvironments, offering new targets for therapeutic intervention.

### 3.4 Spatial metabolomics: are specific microbial metabolites spatially associated with regions of tumour cell invasion or metastasis formation, and how do these metabolites impact cellular behaviour?

Specific microbial metabolites have been increasingly recognised for their role in modulating the TME, particularly in regions of tumour cell invasion and metastasis formation. These metabolites, such as short-chain fatty acids (SCFAs), bile acids and other microbial by-products, can influence tumour cell behaviour by altering cellular metabolism, immune responses and the extracellular matrix, all of which are crucial for metastatic progression (Zhou et al., 2022). Spatial metabolomics techniques, including mass spectrometry imaging (MSI), enable the high-resolution mapping of metabolites within tissues, allowing for the identification of spatial associations between specific microbial metabolites and regions of tumour cell invasion. MSI provides a powerful tool to visualise the distribution of these metabolites in both targeted and untargeted approaches, revealing how microbial metabolites may accumulate in areas of active invasion or metastasis. By mapping metabolic heterogeneity within the TME, spatial metabolomics offers valuable insights into how microbial-derived metabolites can impact tumour cell plasticity, as well as their ability to invade surrounding tissues. Understanding these metabolic interactions provides a foundation for new therapeutic strategies aimed at targeting microbial metabolic pathways in order to prevent or disrupt metastasis.

#### 3.4.1 Mass spectrometry-based methods

Mass spectrometry-based methods like Matrix-Assisted Laser Desorption/Ionisation (MALDI), Desorption Electrospray Ionisation (DESI), Secondary Ion Mass Spectrometry (SIMS), Laser Ablation–Inductively Coupled Plasma–Mass Spectrometry (LA-ICP-MS) and Imaging Mass Cytometry (IMC) are integral to spatial metabolomics, allowing for the precise mapping of metabolites within biological tissues. These techniques enable the high-resolution localisation of metabolites, crucial for unravelling the spatial complexity of biochemical processes in tissue architecture.

In microbiology, MALDI-MSI could play a significant role in visualising metabolites involved in microbial and host-pathogen dynamics, either in 2D or in 3D, providing insights into biofilm and microbial mat formation, chemical characterisation, drug distribution (e.g. antimicrobials distribution within microbial biofilms (Shen et al., 2024)) and effects (Feucherolles and Frache, 2022; Zou et al., 2022; Burguet et al., 2024; Kuik et al., 2024; Vats et al., 2024). Blanc and colleagues’ work highlighted the distribution of the phosphatidylinositol mannosides species and phosphatidylinositol, largely found in *Mycobacterium* spp. lipids fingerprint, observed by MALDI MSI, which shared the same shape around the granuloma cavity, matching the *Mycobacterium* distribution demonstrated by antibody-labelling (Blanc et al., 2018). While the current spatial resolution of commercialised MALDI MSI devices could go down by 5 µm, new demonstrators highlighted the possibility of going down the micrometre scale, making it suitable for potential single-cell analysis in complex biological systems (Kompauer et al., 2016).

Likewise, atmospheric-pressure MALDI MSI, DESI-MSI offers the advantage of a simplified procedure of sample preparation and an ambient ionisation, making it suitable for the rapid spatial analysis of intact biological samples. Microbial imprints, cross-section, suspension and extraction, could be directly analysed to monitor the exchange of metabolites or production of natural products (Watrous et al., 2010; Parrot et al., 2018). Interestingly, the direct visualisation of living bacterial colonies and biofilms, i.e. *Shewanella oneidensis*, *Bacillus subtilis* and *Streptomyces coelicolor*, has been performed by nanospray DESI (nanoDESI) (Watrous et al., 2013). Along the same lines, the rapid detection and identification of bacteria directly in human colorectal tissues, based on DESI-MSI, was reported (Chen et al., 2024). After developing a database containing 3274 bacterial strains and 232 bacterial species using taxon-specific markers, i.e. conserved small metabolite-based markers capable of identifying specific bacterial taxa based on shotgun rapid evaporative ionisation mass spectrometry (REIMS), DESI-MSI was directly used *in situ* to detect a wide range of bacterial biomarkers, including *B. fragilis* and *F. nucleatum*, known to play a key role in the occurrence and the progression in CRC (Strittmatter et al., 2014; Chen et al., 2024). While the study focused on tumour tissue, this type of approach might be considered for metastasis studies. However, it is worth noting that while rapid, DESI or nanoDESI present a critical spatial resolution (ca. 50-200 µm for DESI and <12 µm for nanoDESI) to perform single cell analysis in metastasis.

IMC combines mass spectrometry with high-resolution imaging, enabling the detailed study of microbial populations at the single-cell level (Glasson et al., 2023). IMC can probe up to 40 antigens and transcripts simultaneously in a single experiment, using metal-labelled antibodies, at an attractive spatial resolution (e.g. 1 µm for the *Hyperion+ Imaging System* from Standard Biotools) and hence, is becoming increasingly popular in the growing field of spatial biology. As an example, Feng and colleagues developed an IMC approach for investigating breast cancer tumoural microbiome interactions, using specific tagged labels for localising gram-positive and -negative bacteria and mammalian cells within the fixed tissue (Feng et al., 2022). Kuett and colleagues took the experiment a step further by mapping the cancer breast TME three-dimensionally by combining 152 IMC images, resulting in a voxel of 304x488x652 μm^3^, and enabling metabolic interactions to be studied at high spatial resolution (Kuett et al., 2022). Nevertheless, such a targeted approach requires time-consuming acquisition for volume imaging. Here, IMC could be used to enable the simultaneous co-localisation of immune and bacterial cells in metastasis at a single cell resolution.

SIMS, especially nanoSIMS, offers nanoscale imaging (down to 50 nm) to analyse the elemental and isotopic composition of microbial cells (Nuñez et al., 2018). By sputtering secondary ions from the sample with a focused ion beam and analysing them via mass spectrometry, NanoSIMS could provide detailed insights into microbial metabolic activities and interactions at a single cell level, by imaging natural elemental composition or isotopic distribution, in both cells and spores, and their behaviour in different environmental conditions (Behrens et al., 2008; Ghosal et al., 2008; McGlynn et al., 2015; Chadwick et al., 2019; Pett-Ridge and Weber, 2012). To our knowledge, such methodology has never been directly applied to specifically investigate the microbiome related to either the tumoural or the metastasis microenvironment. However, several studies highlighted its utility individually or combined with other imaging modalities (e.g. FISH and a FISH-based approach) for microbial metabolic activities, such as ^15^N assimilation, ^13^C tracking, sulphur cycling, or biofilm characterisation (Gao et al., 2016). For instance, the anabolic activity of *Geobacter sulfurreducens* biofilms was measured to better understand the phenomenon of microbial conductivity (Chadwick et al., 2019). In the same way, SIMS equipped with a time-of-flight analyser (TOF-SIMS) or an Orbitrap mass analyser (Orbi-SIMS) enables metabolite (e.g. bacterium-derived metabolites and antimicrobials like ciprofloxacin) analysis with precise subcellular localisation in complex matrices, such as native-sate biofilms (Davies et al., 2017; Akbari et al., 2023; Kern et al., 2024). However, a significant drawback of the TOF analyser is its excessive fragmentation during ionisation and its limited mass resolving power (m/Δm ~30,000), which complicates molecule identification and reduces the amount of information obtainable about the metabolic landscape. In contrast, the Orbitrap mass analyser offers the advantages of high mass resolution (>240,000 at m/z 200), high mass accuracy (<1 ppm) and high spatial resolution (down to 2 µm). Kern et al. have developed a workflow combing Orbi-SIMS with fluorescent imaging and histological staining, allowing metabolic segmentation with simultaneous cell identification in the tumour microenvironment (segmentation of tumour cells, immune cells, and stromal areas) (Kern et al., 2024).

Like other elemental methods, LA-ICP-MSI might be used to perform an elemental pre-screening of intact biological tissues at a micrometre level (1-100 µm), as it provides the atomic distribution across the sample surface by using an UV laser. Such an approach has already been investigated to evaluate the elemental distribution (e.g. Mg, Fe, Zn, Ca) and organisation in bacterial colonies or in microbial contaminated tissue abscesses (Corbin et al., 2008; Latimer et al., 2009). More recently, Cassat and colleagues developed a high-resolution imaging platform to investigate the associated alterations of *S. aureus*-triggered infections in mice (Cassat et al., 2018). Here, LA-ICP-MSI was used to follow the distribution of elements, such as calcium, copper, magnesium, manganese, phosphorous, zinc and iron, on adjacent tissues, to highlight the metal starvation responses during *Staphylococcus* infection. Interestingly, in cancer research, such a method has been used extensively for imaging the interaction between anticancer drugs and the cancer cells (Ma and Fernández, 2024). To our knowledge, such an approach has not been used yet to explore the close interaction between metastasis microbiome and its surroundings environment. It might be interesting to evaluate the directly impact of anticancer drugs on the metastasis or on the oncosphere.

In this sense, mass spectrometry-based methodologies combined with other described bulk or spatial omics-powered methods might be a game changer for enhancing our knowledge regarding metastasis microbiome metabolism pathway, through a temporal design experiment at different cancer progression stages, or for the rapid *in situ* detection and identification of bacterial markers.

#### 3.4.2 Fluorescent-based methods

In spatial metabolomics, fluorescent-based methods involve labelling metabolites with fluorescent dyes to visualise their distribution within tissues. This approach allows the real-time tracking and mapping of metabolites using fluorescence microscopy, offering insights into metabolic processes, interactions and tissue organisation. It is especially valuable for studying dynamic biochemical changes and the molecular basis of physiological and pathological conditions. In microbiology, such an approach was investigated for resolving the challenge of understanding the host-commensal interactions within an anaerobic intestinal environment (Geva-Zatorsky et al., 2015). By using metabolic oligosaccharide engineering and bioorthogonal click chemistry (MOE-BCC) to label anaerobes *in vivo* within the mouse gut, including *Bacteroides fragilis*, authors were able to assess distribution and colonisation preferences. Overall, metabolic labelling might be suitable for better understanding the microbial dissemination from the tumour to the metastasis localisation.

### 3.5 Spatial multimodal-omics imaging: how do microbial interactions and metabolic activity within anatomical structures influence metastasis progression?

Considered individually, previously introduced methods are not able to characterise host-metastasis metabolite crosstalks comprehensively, as they either (1) do not have the spatial resolution to reach single-cell size for metabolic interactions (e.g. MALDI MSI), (2) are not able to explain complex heterogeneity in biological ecosystems (e.g. FISH) or (3) do not provide a holistic and non-targeted view of metabolic interactions (e.g. IMC), which further limits our understanding of complex metabolic interactions between the microbiome and the metastasis microenvironment. To close this gap, a tailor-made *in situ* spatial metabolomics workflow needs to be developed, based on the combination of several imaging modalities.

#### 3.5.1 2D Combination of modalities

Combining different 2D MSI modalities with complementary techniques such as LC-MS, H&E staining and RNA scope offers a promising strategy to address key questions regarding microbial and metabolic interactions within metastatic environments. Such an approach has already enabled the study of metabolic and lipidomic variations at the single-cell level in human or mouse liver tissues (Tian et al., 2024). Although this approach is not currently utilised in microbiome research, it presents a promising avenue for exploration.

Integrating MSI with fluorescence-based imaging methods, like FISH and autofluorescence, has been reported in various studies, and has proven useful in mapping microbial distributions and metabolic alterations within infected tissues (Good et al., 2024). For instance, Guiberson and colleagues investigated intestinal tissues infected with *Clostridium difficile* using MALDI MSI, elemental imaging and autofluorescence, observing significant lipid modulation during infection (Guiberson et al., 2022). Parrot and colleagues employed a combination of FISH and DESI to analyse the microbiome and metabolome of brown algae *Fucus vesiculosus* (Parrot et al., 2019). These examples underscore how integrated modalities can resolve microbial and host metabolic interactions spatially, which may parallel microbial involvement in metastasis.

While previous attempts to combine MALDI MSI and FISH on a single tissue section were unsuccessful due to the MALDI laser’s destructiveness, the development of a spatial metabolomics pipeline named metaFISH now allows for the simultaneous imaging of host-microbiome symbioses and their metabolic interactions (Geier et al., 2020; Bourceau et al., 2023). This pipeline offers a culture-independent approach to linking metabolic phenotypes with community members *in situ*, serving as a powerful tool for microbiologists across disciplines. MetaFISH is especially relevant to studying metastasis as it offers a culture-independent method to link microbial presence with host tissue alterations, facilitating the examination of microbiome-tumour crosstalk at the metabolic level.

While previous modalities mainly target the molecular information, it is worth adding elemental imaging modalities to the workflow, as they are both complementary. Such a workflow was discussed and presented by Karst’group (University of Münster, Germany) during the second annual conference on Mass Spectrometry Imaging and Integrated Topics (9–12 September 2024 in Münster, Germany) for biological samples. The multimodal bioimaging approach should rely on both elemental (e.g. micro x-ray fluorescence and LA-ICP-MS) and molecular (e.g. MALDI MSI and QCL-IR microscopy) modalities, on a single tissue sample, as they validate each other and offer complementary insights.

However, as biological interactions are inherently three-dimensional, the transition to 3D approaches would further enhance the understanding of complex metastasis microbiome interactions *in situ*.

#### 3.5.2 From 2D to 3D

Transitioning from 2D to 3D imaging approaches offers a promising avenue to deepen our understanding of the metabolic and anatomical characteristics within complex microbiological ecosystems like primary tumours or the metastasis microenvironment. While many studies have traditionally focused on correlating metabolic distributions in a 2D manner, a shift towards a 3D approach provides an opportunity to glean additional insights into tissue functions and the dynamics of the metastasis microbiome.

One strategy involves integrating bulk omics-powered data, such as mass spectrometry and microbial 16S rRNA amplicon sequences. For instance, Bouslimani et al. demonstrated a method to visualise the chemical composition and microbial community composition of the human skin surface through 3D topographical maps, utilising mass spectrometry data and 16S sequencing, thereby shedding light on the modulation of the skin microbiome and its implications for human health. Another approach entails stacking consecutive images of a single modality to construct 3D reconstructions. For instance, 3D-MALDI MSI was employed to analyse colon cancer-fibroblast co-culture spheroids, yielding detailed maps of metabolic distributions that reflect tissue architecture (Cordes et al., 2021; Iakab et al., 2022).

Another approach combines 2D MSI with advanced 3D imaging workflows such as the chemo-histo-tomography (CHEMHIST) workflow. By integrating, MALDI MSI, micro-computed tomography (micro-CT), FISH and brightfield microscopy, CHEMIST enables the exploration of the relationship between anatomic structure and metabolic function in symbiotic animals, showcasing the potential for the detailed visualisation of microbial interactions and metabolites (Geier et al., 2021). Applied to the metastasis microbiome, a multimodal imaging pipeline combining MALDI MSI, FISH, antibody-based imaging, micro-CT and spatial transcriptomics can uncover the anatomical and metabolic relationships between microbiome constituents and the tumour environment, providing key insights into microbial contributions and functions to metastasis progression.

By harnessing advanced imaging techniques, researchers can address critical needs in microbiome research, including analysing microbial interactions, determining microbial diversity, managing microbiomes and identifying microbial dark matter, as pre-visualised by Biteen and colleagues in 2016 (Biteen et al., 2016). This aligns with recent initiatives such as the CartoHostBug project (https://www.embl.org/news/science/embl-scientists-receive-prestigious-erc-synergy-grants/), which aims to study the spatial architecture of microbiome-host crosstalk in the human gut using novel imaging approaches, spatial transcriptomics technologies and computational modelling techniques.

### 3.6 Interpretation of imaging data in the context of microbiome

To advance our understanding of microbial metabolite interactions within metastatic tumours, it is critical to address the current technical limitations of spatial biology methods. Certain techniques face limitations inherent to their technology, exemplified by MALDI MSI’s potential inadequacies in covering microbial metabolites due to the inefficient ionisation of specific biomolecules (Feucherolles and Frache, 2022). Traditional single laser MALDI methods often yield mass spectra dominated by abundant or easily ionisable analyte molecules, thereby overlooking low-abundance or poorly ionisable molecules, which may be vital in metabolic pathways (Karas et al., 2000; Niehaus et al., 2019). This gap restricts our ability to explore how specific low-abundant microbial metabolites spatially associate with metastasis and impact cellular behaviour.

To mitigate this challenge, innovative approaches like post-ionisation have emerged to enhance ion yield without necessitating additional sample preparation steps. During post-ionisation, a second MALDI-like ionisation event occurs, interacting with the already desorbed particle plume. For instance, Brockmann and colleagues applied post-ionisation techniques to examine microbial communities cultured on polyamide membranes and investigate the inhibition of *P. aeruginosa* by a β-lactam antibiotic (Brockmann et al., 2021). Through overlay images, distinct structures were revealed, showcasing the high abundance of key molecules integral to the quorum-sensing machinery of *P. aeruginosa* proximal to the inhibition zone. The implementation of post-ionisation offers a promising avenue for improving the accuracy of chemical communication analysis within microbial communities by boosting the ion yield of essential biomolecules.

In addressing challenges related to ion yield in imaging techniques, several other strategies have been proposed to maximise the signal detection for specific metabolites or lipids. Firstly, the selection of the matrix plays a crucial role in optimising signal detection for specific metabolites or lipids. For instance, Feenstra and colleagues introduced a multiple matrix MSI approach targeting dual polarities alongside tandem MSI to tackle low metabolite coverage, despite limitations persisting in identifying metabolites with low ionisation energies (Feenstra et al., 2015). Similarly, Angerer and colleagues explored various MALDI matrices to identify the most suitable one for lipid detection (Angerer et al., 2022). However, there is currently a lack of research investigating the optimal matrix for detecting microbial components in MALDI MSI. Adapting and expanding such matrix optimisation strategies could enhance the detection of microbial metabolites. Moreover, sample preparation optimisation techniques, including washing with different solvent and buffer combinations, and recrystallisation, have been employed to improve the detection of specific metabolites such as proteins and lipids (Seeley et al., 2008; Wang et al., 2011; Angel et al., 2012). Additionally, on-tissue chemical derivatisation has emerged as an alternative method to enhance ionisation efficiency. For example, Kaya and colleagues demonstrated the effectiveness of derivatisation reagents in tagging molecules containing carboxylic acid and aldehydes (Kaya et al., 2023). In this sense, there are commercial kits, i.e. *Tag-ON*™, for the on-tissue chemical derivatisation of phenolic hydroxyl and amine functional groups to visualise endogenous neurotransmitters and metabolites (Shariatgorji et al., 2019). However, it is imperative to ensure that such modifications to the sample do not compromise the spatial integrity of the microbial community or interfere with other imaging modalities.

In addition to wet-lab methodologies, leveraging dry-lab techniques offers valuable insights into overcoming various challenges, particularly in enhancing imaging spatial resolution and addressing issues related to multimodal imaging. Spatial resolution limitations pose a significant hurdle, especially when comparing or co-registering results from modalities with disparate spatial resolutions. Techniques like MALDI MSI typically exhibit spatial resolutions of around 15 µm per pixel, even if in-house demonstrators allow for single-cell resolution at 1.4 µm pixel size (Kompauer et al., 2016). Spatial transcriptomics (ST) solutions, both current and upcoming, typically offer resolutions ranging from 0.5 µm to 55 µm.

As spatial omics platforms generate increasingly high-dimensional data across genomics, transcriptomics, proteomics, and metabolomics, artificial intelligence (AI) methods, particularly AI-powered multimodal (deep) learning, have emerged as a critical tool for data integration and interpretation in cancer research (Boehm et al., 2022; Rajdeo et al., 2024). Multimodal AI tools (e.g SOmicsFusion) facilitates the co-registration of data from multiple imaging modalities, such as spatial transcriptomics and mass spectrometry imaging-mediated spatial metabolomics, enabling potential precise mapping of microbial, hosts signals and co-expression patterns within the TME (Guo et al., 2024). These models can uncover patterns of microbial influence on metastatic niches that may not be discernible through single-modality analyses alone. For instance, data fusion workflow, mainly AI-driven, provide a promising paradigm for improving imaging spatial resolution (Van De Plas et al., 2015). By combining techniques with high chemical information but poor spatial resolution (e.g. MALDI MSI) with those possessing low chemical information but high spatial resolution (e.g. TOF-SIMS, microscopy images) through computational tools, sharper ion images with enhanced spatial resolution and chemical specificity can be achieved (Borodinov et al., 2020; Liang et al., 2024). For instance, recent studies have introduced multi-modal image fusion workflows incorporating MALDI imaging mass spectrometry and microscopy, using AI models, i.e. two-dimensional convolutional neural network and random forest, to predict intensity values and patterns while enhancing spatial resolution (Liang et al., 2024). At the end, the original MALDI image was acquired at 25 µm, and the fusion-based image was approximately 2.5 µm, i.e. a ten-fold zoom, suggesting their potential in resolving microbiome-host crosstalk at metastatic fronts.Despite the promise of multimodal imaging approaches, challenges such as the co-registration of imaging datasets, preservation of spatial sample integrity and interpretation of multimodal data, persist. Future efforts, aided by new AI-powered methods, will be pivotal in unravelling complex relationships between data generated by various imaging methods, leading to a more comprehensive understanding of biological systems and pathways (Neumann et al., 2020). Looking ahead, the integration of multimodal biomedical artificial intelligence and imaging technologies with other modalities like biosensors, and genome and microbiome sequencing holds significant promise in advancing our understanding of tumour and metastasis biology (Acosta et al., 2022).

## 4 Conclusion

Much like the multifactorial and multicompartmental nature of cancer metastasis, the methods available to explore and study the disease are just as varied and complex. In this review, we have delved into the relevance of the microbiome in cancer metastasis progression, emphasising its potential role in modulating the TME, immune responses and metabolic pathways. A growing body of research has established that the microbiome plays a crucial role in various stages of the metastatic cascade. The field has progressed beyond correlation studies, increasingly focusing on uncovering mechanistic insights. However, many of these findings remain limited to single-microbe, single-mechanism insights, which do not fully capture the complexity of the physiological context nor the disease. We have also examined a range of spatial omics techniques that are currently being used to address critical questions within the field. These questions include the spatial organisation of microbes within metastatic tissues, the microbiome’s impact on host gene expression and the way in which microbial interactions influence the immune and metabolic landscapes of the TME. However, as promising as these technological advancements appear, they introduce significant challenges in terms of data interpretation and the pinpointing of specific bacterial mechanisms. The sheer volume and complexity of data generated by omics and untargeted methodologies can be overwhelming, often leading to a level of “noise” that makes it difficult to confidently associate specific microbial factors with metastasis. Despite these challenges, we remain optimistic about the future of this field. The integration of spatial omics, multimodal imaging and AI-driven technologies holds immense promise for advancing our understanding of the microbiome’s contribution to metastasis and gaining deeper insights into the intricate relationship between the microbiome and metastatic progression.
